# Clinical utility and tolerability of ketamine as adjunctive rescue therapy for refractory neonatal status epilepticus: a case report of a late preterm infant with multifocal hemorrhagic brain injury

**DOI:** 10.3389/fped.2026.1949800

**Published:** 2026-09-03

**Authors:** Chiara Gulisano, Alessia Pepe, Elisabetta Amadori, Samuele Caruggi, Daniele Caratozzolo, Lino Nobili, Pasquale Striano, Luca Antonio Ramenghi, Andrea Calandrino

**Affiliations:** 1 Department of Neuroscience, Rehabilitation, Ophthalmology, Genetics, Mother and Child Health, School of Medical and Pharmaceuticals, University of Genoa, Genoa, Italy; 2 Neonatal Intensive Care Unit, Department of Maternal and Neonatal Health, IRCCS Istituto Giannina Gaslini, Genoa, Italy; 3 Child Neuropsichiaty Unit, Department of Pediatric Sciences, IRCCS Istituto Giannina Gaslini, Genoa, Italy; 4 Pediatric Neurology and Muscular Diseases, Full Member of ERN epiCARE, Department of Pediatric Sciences, IRCCS Istituto Giannina Gaslini, Genoa, Italy

**Keywords:** continuous video-EEG monitoring, hemorrhagic brain injury, ketamine, neonatal status epilepticus, refractory neonatal seizures

## Abstract

**Background:**

Neonatal status epilepticus (NSE) is a life-threatening neurological emergency associated with high mortality and long-term neurodevelopmental morbidity. Although phenobarbital remains the recommended first-line antiseizure medication (ASM), followed by agents such as phenytoin or levetiracetam, seizure control is not always achieved. Ketamine, a non-competitive N methyl-D-aspartate (NMDA) receptor antagonist, has emerged as a potential rescue therapy for refractory status epilepticus because of its unique mechanism of action and favorable hemodynamic profile. However, evidence supporting its use in neonates remains scarce. Despite increasing recognition of refractory neonatal status epilepticus (RNSE), no universally accepted definition exists. Current recommendations suggest considering RNSE when electroclinical or electrographic seizures persist despite appropriate first- and second-line treatment, highlighting the importance of continuous EEG monitoring.

**Case presentation:**

We report a late preterm male infant with multifocal hemorrhagic brain injury who developed RNSE on the second day of life despite sequential treatment with phenobarbital, levetiracetam and midazolam. Ketamine was associated with disappearance of clinical seizures and persistence of shorter electrographic-only seizures. Complete electrographic seizure cessation occurred after continued ketamine and the subsequent addition of phenytoin. No acute hemodynamic or respiratory adverse events attributable to ketamine were observed.

**Conclusions:**

This case highlights both the potential and the limitations of ketamine in RNSE. Although seizure control cannot be attributed solely to ketamine because of concomitant multimodal therapy, its distinct pharmacological mechanism, favorable cardiovascular profile, and ease of administration make it a feasible and well-tolerated adjunctive treatment option. Continuous EEG monitoring remains essential for evaluating treatment response. Prospective neonatal studies are needed to define the optimal timing, dosing strategies, efficacy, and long-term safety of ketamine in RNSE.

## 1 Introduction

Neonatal seizures represent the most common seizures across the human lifespan and usually reflect acute symptomatic brain injury resulting from hypoxic-ischemic encephalopathy, intracranial hemorrhage, arterial ischemic stroke, central nervous system infection, metabolic disturbances, or structural abnormalities (1). Regardless of etiology prolonged seizure activity is associated with an increased risk of secondary neuronal injury and adverse neurodevelopmental outcomes. Because a substantial proportion of neonatal seizures are electrographic-only, particularly after antiseizures treatment, continuous video-EEG monitoring is currently considered the gold standard for diagnosis and assessment of treatment response (2). According to the most recent International League Against Epilepsy (ILAE) recommendations, phenobarbital remains the recommended first-line antiseizure medication whereas phenytoin or levetiracetam are used as second-line therapies (2). However, therapeutic options for RNSE remain poorly defined and neonatal specific evidence for third live treatments is limited.

Ketamine, a non-competitive NMDA receptor antagonist, has a distinct mechanism of action from conventional GABAergic antiseizure medications. Its use in prolonged status epilepticus is supported by the increasing contribution of NMDA-mediated glutamergic transmission to seizure maintenance during pharmacoresistance. The rationale may be particularly relevant during the neonatal period, when the immature brain exhibits enhanced glutamatergic signalling and relatively higher NMDA receptor expression compared with later stages of development (3). In addition, its intravenous administration, titratability and relatively favourable cardiovascular profile may offer practical advantages in critically ill neonates (4, 5). Nevertheless, clinical experience in neonatal population remains limited to case reports and small observational studies (6–8), precluding firm recommendations regarding its routine use.

We report the case of a late preterm infant with severe multifocal hemorrhagic brain injury who developed EEG-confirmed RNSE treated with adjunctive ketamine.

This case aims to describe the feasibility and tolerability of ketamine within a multimodal treatment strategy, with particular emphasis on its practical use in the NICU and the role of continuous EEG in assessing treatment response. The case also contributes to the limited clinical experience available in this population and highlights the need for further prospective multicentre studies.

## 2 Case description

A male infant was born at 35 + 4 weeks’ gestation from a dichorionic twin pregnancy complicated by fetal growth restriction of the index fetus, early placental flow insufficiency, and preterm premature rupture of membranes. Maternal infectious screening was unremarkable. Delivery was performed by urgent cesarean section because of abnormal cardiotocography. At birth, the infant was hypotonic, cyanotic, and poorly responsive, requiring brief positive-pressure ventilation followed by continuous positive airway pressure (CPAP) support, with rapid clinical improvement. Apgar scores were 5, 7, and 8 at 1, 5, and 10 min, respectively. Umbilical cord blood gas analysis showed mild metabolic acidosis (pH 7.17, base excess −9.6 mmol/L), with hemoglobin of 20.6 g/dL. Birth weight was 1930g (7th percentile, small for gestational age according to the INeS Charts).

### 2.1 Diagnostic assessment

Cranial ultrasonography performed shortly after birth demonstrated bilateral grade II–III intraventricular hemorrhage associated with mild post-hemorrhagic ventricular dilatation without evidence of rapidly progressive hydrocephalus. On the second day of life, the infant developed recurrent episodes of desaturation associated with hypertonia, raising suspicion of neonatal seizures. Continuous video-electroencephalographic (EEG) monitoring revealed recurrent focal electroclinical seizures arising predominantly from the left temporal region in the setting of a severely abnormal background characterized by discontinuity and multifocal epileptiform discharges. During the following hours, seizure burden progressively increased, with evolution toward multifocal electrographic-only seizures, fulfilling the electroclinical criteria for refractory neonatal status epilepticus. Once clinical stabilization had been achieved and clinical seizures had been controlled, brain magnetic resonance imaging (MRI) was performed. It demonstrated extensive multifocal supra- and infratentorial hemorrhagic injury, including intraventricular, extra-axial, and subpial hemorrhages, together with a hemorrhagic lesion involving the right paravermian and vermian regions, causing compression of the fourth ventricle and early ventricular enlargement. Diffusion-weighted imaging also showed multifocal areas of acute parenchymal injury. Follow-up MRI performed during hospitalization documented progression toward diffuse cerebral injury, including cortical laminar necrosis, evolving white matter damage, enlargement of supratentorial subdural collections, and chronic supra- and infratentorial sequelae. Given the severity and atypical pattern of brain lesions, additional diagnostic investigations were undertaken. Empirical broad-spectrum antibiotic therapy with ampicillin and gentamicin was initiated at birth and subsequently discontinued after inflammatory markers, including C-reactive protein and procalcitonin, and microbiological cultures remained negative. Hematological investigations revealed marked hypofibrinogenemia and prolonged coagulation times (PT and aPTT), requiring multiple transfusions of fresh frozen plasma and fibrinogen supplementation (100 mg/kg), with subsequent resolution of the coagulopathy. Factor XIII, factors II and V, protein C, and protein S levels were within normal ranges. Thrombocytopenia was also present, with a nadir platelet count of 83,000/mm³ on the second day of life, requiring platelet transfusion. Severe anemia developed during the first day of life (hemoglobin 8 g/dL), requiring transfusion of packed red blood cells. Given the severity and multifactorial nature of the clinical presentation, an extensive metabolic work-up was performed, including basic and extended newborn metabolic screening, plasma amino acid chromatography, urine organic acid analysis, acylcarnitine profiling, ammonia, ceruloplasmin, and serum copper levels; all results were within normal limits. A genetic evaluation was also performed because of dysmorphic features, including a broad forehead, mild hypertelorism, thick upper lip, gingival hypertrophy, micrognathia, and a right ear with posterior rotation and abnormal folding. Trio whole-exome sequencing (WES) was subsequently performed and did not identify any pathogenic variants.

### 2.2 Therapeutic intervention

**Figure 1:**
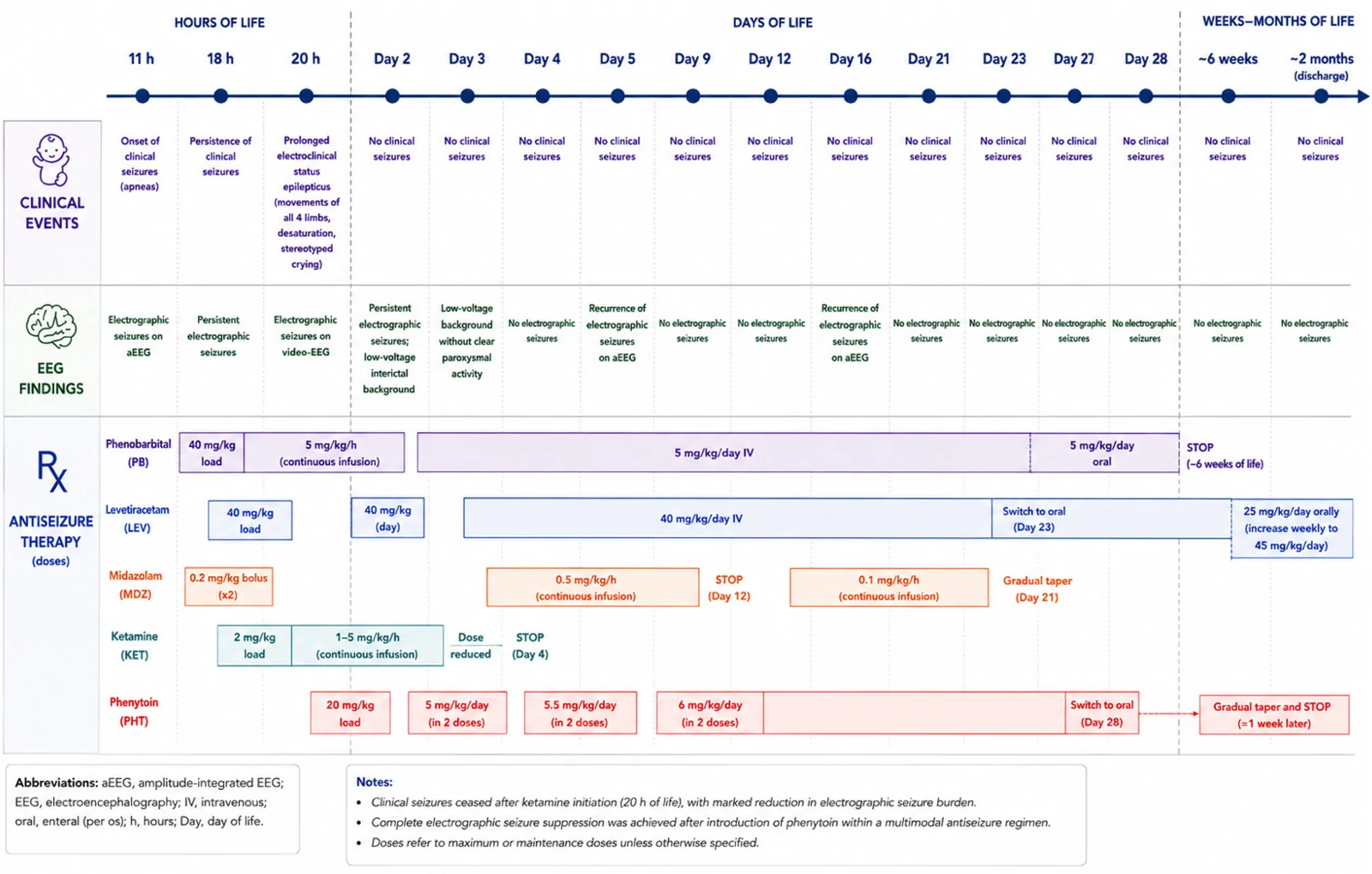
Timeline of seizure, EEG findings and antiseizure therapy.

Initial antiseizure treatment consisted of phenobarbital (total dose of 40 mg/kg) followed by levetiracetam (loading dose of 40 mg/kg) and repeated intravenous midazolam boluses (0.1–0.2 mg/kg). Despite sequential escalation of therapy, continuous EEG demonstrated persistent electrographic seizures with a high seizure burden. Ketamine was therefore introduced as adjunctive rescue therapy with an intravenous loading dose of 2 mg/kg followed by continuous infusion, titrated up to 5 mg/kg/day. Following ketamine administration, seizures evolved from electroclinical to exclusively electrographic events, with a concomitant reduction in seizure duration. The background activity evolved to a low-voltage pattern with an absence of normal physiological features, while multifocal epileptiform abnormalities remained superimposed. However, because recurrent electrographic seizures persisted, ketamine was further titrated and stopped at fourth day of life and adjunctive phenytoin was added with a loading dose (20 mg/kg) and continued as maintenance therapy (5 mg/kg/day), together with ongoing midazolam infusion started at four day of life (0.5 mg/kg/h) and gradually decreased until suspension after three weeks. This was followed by a progressive reduction in seizure frequency, ultimately leading to complete electrographic seizure resolution. Phenytoin and phenobarbital were progressively tapered and discontinued by approximately 2 months of age. At discharge, antiseizure treatment was continued exclusively with oral levetiracetam, initiated at 25.6 mg/kg/day and planned to be increased weekly to a maintenance dose of 45 mg/kg/day (Figure 1).

### 2.3 Follow-up and outcomes

Following initiation of ketamine and subsequent escalation of antiseizure therapy, clinically apparent seizures were no longer observed. Continuous video-EEG monitoring was maintained throughout the acute phase for several days with last registration at two months of life. During continuous infusion of ketamine and midazolam, the EEG background was characterized by diffusely low-voltage activity without further electroclinical seizures. Although a few electrographic-only seizures were still recorded during the early phase after ketamine initiation, no additional electrographic seizures were observed following completion of multimodal antiseizure therapy. As ketamine and midazolam were gradually tapered, the EEG background progressively evolved toward a normal-voltage pattern. However, background organization remained initially abnormal, with persistent discontinuity and superimposed multifocal epileptiform abnormalities, consistent with the severity of the underlying structural brain injury. Once continuous EEG monitoring confirmed sustained seizure freedom, with no further electroclinical or electrographic seizures, continuous monitoring was discontinued. Thereafter, follow-up was performed with serial conventional EEG recordings, which consistently demonstrated persistent background abnormalities and multifocal epileptiform discharges but no evidence of seizure recurrence. Follow-up neuroimaging demonstrated gradual evolution of the hemorrhagic lesions toward chronic supra- and infratentorial tissue loss, ventriculomegaly, cortical laminar necrosis, and gradually resolving extra-axial collections. Because of progressive ventricular dilatation, the infant underwent two therapeutic lumbar punctures. No acute cardiovascular and respiratory instability, or other adverse events directly attributable to ketamine were observed during treatment. At discharge, antiseizure therapy had been progressively simplified, with discontinuation of phenytoin while levetiracetam was maintained because of the severity of the underlying brain injury and the high risk of subsequent epilepsy.

## 3 Discussion

Neonatal seizures remain one of the most challenging neurological emergencies encountered in neonatal intensive care, particularly when they evolve into a refractory status epilepticus (9, 10). Although phenobarbital remains the recommended first-line antiseizure medication and phenytoin or levetiracetam are commonly used as second-line therapies, a substantial proportion of neonates continue to experience seizures despite escalating treatment (11). Consequently, management of RNSE often relies on multimodal therapeutic approaches supported by limited evidence (1, 2).

The present case illustrates several clinically relevant aspects of RNSE management. First, it highlights the indispensable role of continuous video-EEG in detecting the transition from electroclinical to predominantly electrographic-only seizures, a well-recognized phenomenon following antiseizure treatment in neonates. Reliance on clinical observation alone would have substantially underestimated seizure burden and might have resulted in premature discontinuation of treatment. Continuous EEG therefore remains the cornerstone for both diagnosis and therapeutic decision-making in neonatal status epilepticus (2).

Second, ketamine was introduced as an adjunctive therapy after failure of conventional antiseizure medications because of its distinct mechanism of action and favourable hemodynamic profile. Rather than replacing established therapies, ketamine may complement GABAergic antiseizure medications by targeting N-methyl-D-aspartate (NMDA) receptor, whose expression is relatively increased in immature brain. This mechanism becomes particularly relevant during prolonged status epilepticus, when progressive internalization of synaptic GABA_A receptors reduces responsiveness to benzodiazepines and barbiturates, while persistent activation of glutamatergic pathways contributes to ongoing seizure activity (3, 12).

Beyond its pharmacological mechanism, ketamine possesses several practical characteristics that may facilitate its use in critically ill neonates. Intravenous administration is straightforward, and continuous infusion allows progressive dose titration according to continuous EEG monitoring (4).

Another important consideration is the hemodynamic profile of ketamine. Unlike barbiturates or high-dose benzodiazepines, ketamine generally preserves systemic blood pressure through sympathetic stimulation and is less frequently associated with clinically significant hypotension. Preservation of cardiovascular stability may be especially valuable in preterm infants, in whom cerebral autoregulation is immature and fluctuations in systemic perfusion may exacerbate ongoing brain injury (6). These characteristics make ketamine a feasible adjunctive option that may be considered within a multimodal treatment strategy for selected neonates with persistent seizures despite conventional therapy.

One of the major strengths of the present case is the availability of prolonged continuous EEG monitoring, allowing accurate interpretation of treatment response. Continuous EEG documented a clinically meaningful change after ketamine: electroclinical seizures ceased and residual seizures became exclusively electrographic and shorter. However, complete electrographic seizure suppression was achieved only after subsequent phenytoin administration while ketamine and other ASMs were continued. Thus, the temporal association supports a possible adjunctive antiseizure contribution of ketamine, but its independent effect cannot be disentangled from phenytoin and the cumulative effects of polytherapy. Accordingly, our findings should be interpreted as supporting the feasibility of ketamine as adjunctive rescue therapy rather than demonstrating efficacy as monotherapy. Moreover, this case reinforces the concept that apparent clinical seizure control should never be considered equivalent to electrographic seizure cessation. Continuous EEG monitoring remains essential for evaluating treatment efficacy, particularly in neonates, in whom electrographic-only seizures frequently account for a substantial proportion of seizure burden.

Safety remains one of the principal concerns regarding ketamine administration during the neonatal period. In the present case, no acute cardiovascular or respiratory instability or other adverse events were clearly attributable to ketamine. This observation is consistent with the limited neonatal literature, in which serious acute adverse effects have been reported infrequently. Nevertheless, currently available evidence is based almost exclusively on isolated case reports, small retrospective case series, and mixed paediatric cohorts including relatively few neonates (6–8) [Table 1]. Consequently, conclusions regarding safety should be drawn cautiously. Important questions remain regarding optimal dosing, pharmacokinetics in preterm infants, potential interactions with concomitant antiseizure medications, and long-term neurodevelopmental outcomes following neonatal exposure.

**Table 1:** Published neonatal cases treated with ketamine for refractory or super-refractory status epilepticus.

Study	GA (wk)	Etiology	Neuroimaging	EEG	Prior ASMs, *n*	Ketamine (line/regimen)	EEG outcome	AEs	Clinical outcome
Tegtmeyer et al., 1995	Term	NKH encephalopathy	None	Continuous BS	2	3rd: IV CI 0.1 mg/kg/h × 4 wk → PO 1 mg/kg/day	Transient improvement; rebound	None	Seizure cessation; persistent severe motor impairment
Haider et al., 1996	40	NKH encephalopathy	CT abnormalities	BS	2	3rd: 1 mg BID	NR	None	Seizure-free; spastic quadriplegia
Dhamija et al., 2012	41 + 5	NKH encephalopathy	MRI abnormalities	Asynchronous BS	1	2nd: NR	NR	None	Persistent seizures and severe developmental impairment
Tarocco et al., 2014	34	Lissencephaly/polymicrogyria	NR	EEOE with BS	6	6th: 2 mg/kg bolus → CI 10–24 μg/kg/min	Initial response; rebound	None	Initial seizure control followed by death
Freibauer et al., 2018	38	KCNQ2-DEE	MRI abnormalities	Discontinuous BS	3	4th: NR	No improvement	None	Death
Huntsman et al., 2019	39 + 1	HIE + hemorrhagic stroke	MRI abnormalities	Frequent electrographic seizures	4	5th: CI 0.5–1.5 mg/kg/h	Seizure control	None	Seizure-free; normal neurological examination
Mandal et al., 2022	NR	Partial hemimegalencephaly	MRI: HME	NR	4	6th: NR	Complete seizure resolution	None	Favorable seizure control
Pin et al., 2022	40 + 3	HIE	MRI abnormalities	Multifocal seizures	7	7th: CI 10–100 μg/kg/min × 8 days	Seizure control	None	Death
Present case	35 + 4	Multifocal hemorrhagic brain injury	Extensive supra-/infratentorial hemorrhage	Recurrent focal seizures on discontinuous background	4	5th: 2 mg/kg IV bolus → CI up to 5 mg/kg/day	Complete seizure control	None	Seizure resolution; persistent structural brain injury

ACC, agenesis of the corpus callosum; AE, adverse event; ASM, antiseizure medication; BID, twice daily; BS, burst-suppression; CBZ, carbamazepine; CI, continuous infusion; CT, computed tomography; DEE, developmental and epileptic encephalopathy; EEOE, early epileptic epileptic encephalopathy; GA, gestational age; HIE, hypoxic-ischemic encephalopathy; HME, hemimegalencephaly; IV, intravenous; LEV, levetiracetam; MDZ, midazolam; MRI, magnetic resonance imaging; NKH, non-ketotic hyperglycinemia; NR, not reported; PB, phenobarbital; PHT, phenytoin; PO, oral; TPM, topiramate. Neuroimaging findings: Haider et al.: bilateral frontoparietal hypodensities on CT. Dhamija et al.: agenesis of the corpus callosum, immature sulcation, and elevated glycine peak on MR spectroscopy. Freibauer et al.: patchy deep white matter T2 hyperintensities. Huntsman et al.: large subacute infarction with intracranial hemorrhages. Mandal et al.: partial parieto-occipital hemimegalencephaly. Pin et al.: bilateral cortical/subcortical and thalamic diffusion restriction. Present case: extensive multifocal supra- and infratentorial hemorrhagic injury involving intraventricular, extra-axial, and subpial hemorrhages, with a right paravermian/vermian hemorrhagic lesion causing fourth ventricular compression and early ventriculomegaly. Prior ASMs: Tegtmeyer et al.: diazepam, midazolam. Haider et al.: phenobarbital, phenytoin. Dhamija et al.: phenobarbital. Tarocco et al.: phenobarbital, phenytoin, midazolam, levetiracetam, propofol, carbamazepine. Freibauer et al.: phenobarbital, phenytoin, midazolam. Huntsman et al.: phenobarbital, phenytoin, midazolam, topiramate. Mandal et al.: phenobarbital, phenytoin, midazolam, topiramate. Pin et al.: phenobarbital, levetiracetam, phenytoin, midazolam, lidocaine, pyridoxine, and thiopental. Present case: phenobarbital, levetiracetam, midazolam, and phenytoin.

Overall, this case adds to the limited clinical experience with ketamine in neonatal refractory status epilepticus. Rather than supporting specific therapeutic recommendations, it highlights the feasibility and tolerability of incorporating ketamine into a multimodal treatment strategy under continuous EEG monitoring and underscores the need for prospective multicentre studies to better define its role, safety profile and optimal use in neonatal intensive care unit.

## 4 Conclusions

Our case contributes additional evidence supporting the feasibility of ketamine administration in a late preterm infant with severe structural brain injury and EEG-confirmed refractory status epilepticus. Importantly, the prolonged EEG documentation available in this patient allowed a more accurate assessment of treatment response than has been reported in several previous neonatal cases. At the same time, our findings emphasize that ketamine should be viewed as part of a multimodal therapeutic strategy rather than as an isolated intervention.

## 5 Limitations and future implications

Several limitations should be acknowledged. First, this report describes a single patient, precluding any inference regarding treatment efficacy or generalizability. Second, the underlying extensive hemorrhagic brain injury strongly influenced both seizure burden and neurological prognosis. Third, multiple antiseizure medications were administered in rapid succession, making it impossible to determine the independent contribution of ketamine to seizure control. Finally, long-term neurodevelopmental follow-up is not yet available and therefore the potential impact of ketamine on later neurological outcome cannot be assessed.

Despite these limitations, this case adds clinically relevant information to the limited evidence supporting ketamine use in neonatal refractory status epilepticus. Future prospective multicenter studies are needed to define the optimal timing of ketamine introduction, appropriate dosing strategies for term and preterm neonates, pharmacokinetic and pharmacodynamic characteristics during the neonatal period, and long-term neurodevelopmental safety. Until such evidence becomes available, ketamine should be considered an adjunctive rescue therapy reserved for carefully selected neonates managed in centers with expertise in continuous EEG monitoring and neonatal neurocritical care.

## Data Availability

The raw data supporting the conclusions of this article will be made available by the authors, without undue reservation.
